# Molecular epidemiology of Chinese Han deaf patients with bi-allelic and mono-allelic *GJB2* mutations

**DOI:** 10.1186/s13023-020-1311-2

**Published:** 2020-01-28

**Authors:** Xiaoyu Yu, Yun Lin, Jun Xu, Tuanjie Che, Lin Li, Tao Yang, Hao Wu

**Affiliations:** 1 Department of Otorhinolaryngology-Head and Neck Surgery, Shanghai Ninth People’s Hospital, Shanghai Jiao Tong University School of Medicine, Shanghai, China; 2 Ear Institute, Shanghai Jiao Tong University School of Medicine, Shanghai, China; 3 Shanghai Key Laboratory of Translational Medicine on Ear and Nose Diseases, Shanghai, China; 4 Key Laboratory of Functional Genomic and Molecular Diagnosis of Gansu Province, Lanzhou, 730030 China; 5 Laboratory of Precision Medicine and Translational Medicine, Suzhou Hospital Affiliated to Nanjing Medical University, Suzhou Science and Technology Town Hospital, Suzhou, 215153 China

**Keywords:** Hearing loss, *GJB2*, Mutation screening, Epidemiology, Next generation sequencing

## Abstract

**Background:**

Recessive mutations in *GJB2* is the most common cause of genetic hearing loss worldwide. The aim of this study is to determine the spectrum and frequency of *GJB2* variants in Chinese Han deaf patients and to investigate the underlying causative genes in patients with mono-allelic *GJB2* mutations.

**Methods:**

We analyzed the mutation screening results of *GJB2* in 1852 Chinese Han probands with apparently autosomal-recessive hearing loss in our laboratory. Targeted next-generation sequencing of 139 known deafness-related genes were performed in 44 probands with mono-allelic *GJB2* mutations.

**Results:**

Bi-allelic *GJB2* mutations was identified in 25.65% of patients, in which the c.235delC (p.L79Cfs*3) mutation is the most frequent cause for both severe-to-profound (84.93%) and mild-to-moderate hearing loss (54.05%), while the c.109G > A (p.V37I) mutation is another frequent cause for mild-to-moderate hearing loss (40.54%). In 3.89% of patients only one mutant allele can be identified in *GJB2*. Targeted next generation sequencing in 44 such probands revealed digenic heterozygous mutations in *GJB2/GJB6* and *GJB2/GJB3* as the likely pathogenic mechanism in three probands. In 13 probands, on the other hand, pathogenic mutations in other deafness-associated genes (*STRC, EYA1, MITF, PCDH15, USH2A, MYO15A, CDH23, OTOF, SLC26A4, SMPX,* and *TIMM8A*) can be identified as the independent genetic cause, suggesting that the mono-allelic *GJB2* mutations in those probands is likely co-incidental.

**Conclusions:**

Our results demonstrated that *GJB2* should be a primary target for mutation screening in Chinese Han deaf patients, and those with mono-allelic *GJB2* mutations should be further screened by next generation sequencing.

## Introduction

Hearing loss is a heterogeneous disorder that affects language acquisition and social skill development in children. It is estimated that 50%~ 60% cases of hearing loss have a genetic etiology [1]. To date, there have been over 100 genes identified to cause non-syndromic hearing loss and over 700 genetic syndromes described with features of hearing loss. Despite this, mutations in a single gene *GJB2* (OMIM 121011) account for a large proportion of non-syndromic hearing loss in most populations worldwide [2].

The *GJB2* gene codes for a gap junction protein connexin-26 (Cx26), which is essential for the physiological function of supporting cells in the cochlea [3]. About 200 *GJB2* pathogenic mutations have been reported so far [4]. A number of missense mutations may lead to autosomal dominant non-syndromic hearing loss DFNA3 and autosomal dominant syndromic hearing loss associated with hyperproliferative epidermal disorders [5, 6]. On the other hand, a majority of *GJB2* mutations are inherited in a recessive form and lead to non-syndromic hearing loss DFNB1. The mutation spectrum of *GJB2* and the frequencies of these mutations vary greatly across different ethnic groups [2, 7], and the Chinese population has a quite distinct spectrum of *GJB2* mutations from other populations [8]. With China having approximately one fifth of the world’s population, evaluating the molecular epidemiology of *GJB2* mutations in Chinese deaf patients has important implications in guiding genetic testing for deafness. In the present study, we analyzed the *GJB2* mutation screening results and audiometric data of 1852 Chinese Han deaf probands to determine its *GJB2* mutation spectrum and genotype-phenotype correlation.

In addition, previous mutation screening of *GJB2* in deaf patients revealed that a substantial number of them carried only one mutant allele [2, 9, 10]. The allele frequency of *GJB2* mutations in heterozygous patients was significantly higher than expected in the general population. Possibly other mutations, either within the DFNB1 locus or in other unlinked genes, could contribute to the hearing loss in patients with mono-allelic *GJB2* mutations. To this end, this study also used targeted next-generation sequencing (NGS) to detect single nucleotide variants, small insertions and deletions (indels) and copy number variations (CNVs) of 139 known deafness-related genes in 44 patients with mono-allelic *GJB2* mutations. The results would provide important information for genetic testing and counselling, especially for those with mono-allelic *GJB2* mutations.

## Materials and methods

### Patients

We reviewed the records of patients with sensorineural hearing loss who received genetic testing for deafness in our laboratory in Shanghai Ninth People’s Hospital, Shanghai Jiao Tong University School of Medicine. Included in this study were patients with bilateral, non-syndromic, sensorineural hearing loss. A total of 1852 unrelated deaf probands, 979 males and 873 females, were analyzed for *GJB2* testing and audiological examination results. The familial cases were compatible with an autosomal-recessive inheritance and the rest cases were sporadic. The ages of the subjects ranged from 2 months to 68 years, with the median age of 12 years old. All subjects were of Chinese Han ethnicity. The severity of hearing loss was classified based on the better hearing ear as mild (21~40 dB), moderate (41~70 dB), severe (71~ 95 dB), and profound (> 95 dB).

### Ethics statement

A written informed consent was obtained from each subject or their guardians to participate in this study. This study was approved by the Ethics Committee of Shanghai Ninth People’s Hospital, Shanghai Jiao Tong University School of Medicine.

### Mutation analysis of the *GJB2* gene

Genomic DNA was extracted from blood samples using the Genomic DNA Extraction Kit (Tiangen Biotech, Beijing, China). The *GJB2* (NM_004004.5) coding exon (exon 2) and flanking regions as well as the non-coding exon 1 and its flanking splice sites were amplified by polymerase chain reaction (PCR), and the PCR product was then Sanger sequenced in both directions. Sequence data were analyzed using Sequencher 5.4.5, and primer sequences are provided in Additional file 1: Table S4.

### Targeted next-generation sequencing

For library preparation, 2μg genomic DNA was randomly fragmented to 150–200 bp fragments by ultrasound shearing. End-repair, adenylation, adapter ligation and PCR amplification were completed according to the standard Illumina protocol. The amplified DNA was captured with a Deafness-related Gene Panel (WuXi NextCODE, Shanghai, China) designed to capture all exons and splicing sites of 139 deafness genes. Sequencing of the enrichment libraries were then performed on the Illumina HiSeq high-throughput platform.

The raw reads were mapped to the human reference genome (UCSC hg19), and the Sentieon software suite was used to call Single Nucleotide Variants (SNVs) and small insertions or deletions (InDels). Copy number variation detection was carried out with CNVkit [11] and ExomeDepth [12] tools that detect copy number variations based on read depth. The SNVs and InDels were annotated with an in-house developed annotation pipeline developed by WuXi NextCODE using Variant Effect Predictor (VEP) software.

### Variant filtering and interpretation

With the exception of three known common mutations in Chinese Hans, c.235delC and c.109G > A in *GJB2* and c.919-2A > G in *SLC26A4*, an in-house Chinese Han allele frequency database was used to exclude variants with minor mutation frequency (MAF) higher than 0.005 in the general population. ClinVar, OMIM and HGMD database were used to annotate known pathogenic variants. In addition, multiple computational tools (SIFT, Polyphen2, PROVEAN, MutationTaster and PANTHER) were used to predict the functionality of nonsynonymous variants. Segregation analysis was performed when DNA samples from the family members were available. The reported variants and CNVs were validated by Sanger sequencing (primer sequences for PCR amplification are provided in Additional file 1: Table S4).

## Results

### Spectrum and genotype-phenotype correlation of *GJB2* mutations

Sanger sequencing of both the coding and noncoding exon and flanking sites of *GJB2* in 1852 Chinese Han deaf probands identified a total of 47 different mutations. The most frequent variants included four frameshift mutations c.235delC (p.L79Cfs*3, allele frequency 18.25%, 676/3704), c.299_300delAT (p.H100Rfs*14, 2.94%), c.507insAACG (p.A171Efs*40, 0.65%), c.36insG (p.V13Cfs*35, 0.24%) and three missense mutations c.109G > A (p.V37I, 7.88%), c.368C > A (p.T123 N, 0.84%) and c.257C > G (p.T86R, 0.51%) (Table 1). In addition, four dominant mutations c.164C > A (p. T55 N), c.224G > A (p. R75Q), c.223C > T (p. R75W) and c.551G > A (p. R184Q) were identified in seven subjects and fourteen unclassified variants were detected in 51 subjects (Additional file 1: Tables S1&S3).

**Table 1:** Pathogenic or unclassified variants in *GJB2* among 1852 deaf patients

Nucleotide Change	Effect on Protein	Allele counts	Allele Frequency in 1852 deaf patients (%,3704 allele)	Category
Frameshift mutations				
c.235delC	frameshift	676	18.25	pathogenic
c.299_300delAT	frameshift	109	2.94	pathogenic
c.507insAACG	frameshift	24	0.65	pathogenic
c.36insG	frameshift	9	0.24	pathogenic
c.176del16bp	frameshift	7	0.19	pathogenic
c.605ins46	frameshift	7	0.19	pathogenic
c.312del14	frameshift	1	0.03	pathogenic
c.35delG	frameshift	1	0.03	pathogenic
c.443delC	frameshift	1	0.03	pathogenic
c.493insG	frameshift	1	0.03	pathogenic
Nonsense mutation			0.00	
c.9G > A	p. W3*	4	0.11	pathogenic
c.139G > T	p. E47*	5	0.13	pathogenic
c.231G > A	p. W77*	2	0.05	pathogenic
Missense mutations			0.00	
c.109G > A	p. V37I	292	7.88	Pathogenic^a^
c.368C > A	p. T123 N	31	0.84	unclassified
c.257C > G	p. T86R	19	0.51	pathogenic
c.427C > T	p. R143W	7	0.19	pathogenic
c.571 T > C	p. F191 L	5	0.13	unclassified
c.344 T > G	p. F115C	4	0.11	unclassified
c.11G > A	p. G4D	3	0.08	pathogenic
c.224G > A	p. R75Q	3	0.08	pathogenic
c.223C > T	p. R75W	2	0.05	pathogenic
c.389G > T	p. G130 V	2	0.05	pathogenic
c.583A > G	p. M195 V	2	0.05	unclassified
c.164C > A	p. T55 N	1	0.03	pathogenic
c.181A > C	p. K61Q	1	0.03	pathogenic
c.187G > T	p. V63 L	1	0.03	pathogenic
c.77C > T	p. T26I	1	0.03	unclassified
c. 95G > A	p. R32H	1	0.03	pathogenic
c.232G > A	p. A78T	1	0.03	unclassified
c.242 T > G	p. L81R	1	0.03	pathogenic
c.250G > A	p. V84 M	1	0.03	pathogenic
c.253 T > C	p. S85P	1	0.03	pathogenic
c.257C > T	p. T86 M	1	0.03	pathogenic
c.379C > T	p. R127C	1	0.03	pathogenic
c.389G > C	p. G130A	1	0.03	pathogenic
c.107 T > C	p. L36P	1	0.03	unclassified
c.398G > C	p. W133S	1	0.03	unclassified
c.457 G > A	p. V153I	1	0.03	unclassified
c.478G > A	p. G160S	1	0.03	unclassified
c.493 C > T	p. R165W	1	0.03	pathogenic
c.551G > A	p. R184Q	1	0.03	pathogenic
c.551G > C	p. R184P	1	0.03	pathogenic
c.586A > G	p. I196V	1	0.03	unclassified
c.587 T > C	p. I196T	1	0.03	unclassified
c.598G > A	p. G200R	1	0.03	pathogenic
Splice site mutation				
c.-3170G > A	IVS1 + 1 G > A	1	0.03	pathogenic

^a^The p.V37I variant is a pathogenic variant with variable expressivity and incomplete penetrance [13]

Overall, bi-allelic (homozygous and compound heterozygous) pathogenic mutations in *GJB2* were identified in 475 probands (25.65%, Table 2). Among them, c.235delC/c.235delC (227, 47.79%), c.109G > A/c.109G > A (53, 11.16%), c.235delC/c.299_300delAT (86, 18.11%), c.235delC/c.109G > A (20, 4.21%), c.235delC/c.507insAACG (18, 3.79%) and c.235delC/c.257C > G (11, 2.32%) were the most common pathogenic *GJB2* genotypes. These six common genotypes were found in up to 87.37% probands with bi-allelic *GJB2* mutations in our cohort (Table 2).

**Table 2:** Genotypes and phenotypes of 475 deaf probands with bi-allelic *GJB2* mutations

Genotype	Severity of Hearing Loss				Total
	Mild	Moderate	Severe	Profound	
c.235delC /c.235delC	0	11	77	139	227
c.109G > A /c.109G > A	7	8	20	18	53
c.299_300delAT/c.299_300delAT	0	0	3	0	3
c.507insAACG /c.507insAACG	0	0	1	0	1
c.235delC /c.299_300delAT	0	2	34	50	86
c.235delC /c.109G > A	0	5	6	9	20
c.235delC /c.507insAACG	0	1	7	10	18
c.235delC /c.257C > G	0	1	2	8	11
c.235delC /c.605ins46	0	0	1	4	5
c.235delC /c.139G > T	0	0	2	3	5
c.235delC /c.9G > A	0	0	1	2	3
c.235delC /c.427C > T	0	0	1	2	3
c.235delC /c.36insG	0	0	0	3	3
c.235delC /c.598G > A	0	0	0	2	2
c.299_300delAT /c.176del16bp	0	1	0	1	2
c.257C > G /c.507insAACG	0	0	0	2	2
c.36insG /c.176del16bp	0	0	0	2	2
c.235delC /c.223C > T	0	0	1	0	1
c.235delC /c.231G > A	0	0	0	1	1
c.235delC /c.242 T > G	0	0	1	0	1
c.235delC /c.257C > T	0	0	0	1	1
c.235delC /c.389G > C	0	0	1	0	1
c.235delC /c.35delG	0	0	0	1	1
c.235delC /c.176del16bp	0	0	0	1	1
c.235delC /c.312del14	0	0	1	0	1
c.235delC /c.11G > A	0	0	1	0	1
c.299_300delAT /c.-3170G > A	0	0	1	0	1
c.299_300delAT /c.257C > G	0	0	0	1	1
c.299_300delAT /c.427C > T	0	0	0	1	1
c.299_300delAT /c.443delC	0	1	0	0	1
c.299_300delAT /c.605ins46	0	0	1	0	1
c.36insG /c.109G > A	0	0	1	0	1
c.36insG /c.427C > T	0	0	1	0	1
c.36insG /c.507insAACG	0	0	0	1	1
c.9G > A /c.231G > A	0	0	1	0	1
c.257C > G /c.493insG	0	0	0	1	1
c.257C > G /c.299_300delAT	0	0	0	1	1
c.379C > T /c.478G > A	0	0	0	1	1
c.109G > A /c.605ins46	0	0	1	0	1
c.109G > A /c.299_300delAT	0	0	0	1	1
c.109G > A /c.181A > C	0	0	1	0	1
c.109G > A /c.250G > A	0	0	1	0	1
c.109G > A /c.257C > G	0	0	1	0	1
c.109G > A / c.427C > T	0	0	1	0	1
c.176del16bp /c.389G > T	0	0	0	1	1
c.176del16bp /c. 95G > A	0	0	0	1	1
Total	7	30	170	268	475

When analyzing the hearing loss levels in these subjects, we found that 92.21% (438/475) of patients with bi-allelic *GJB2* mutations exhibited severe-to-profound hearing loss (Table 2). The c.235delC mutation alone, was identified in 372 (84.93%) such probands in at least one allele. On the contrary, the c.235delC and c.109G > A mutations are the major causes for the remaining 37 probands with mild-to-moderate hearing loss, accounting for 54.05% (20/37) and 40.54% (15/37) of probands in at least one allele, respectively.

### Additional or alternative causes in patients with mono-allelic *GJB2* mutations

In our cohort, we also found 72 (3.89%) deaf probands carrying only a single recessive pathogenic mutation of *GJB2* (Additional file 1: Table S1, the heterozygous c.109G > A variant is not included in this group due to its incomplete penetrance and high frequency in the general population of Chinese Hans [13]). To elucidate the molecular etiology of the hearing loss in probands with mono-allelic *GJB2* mutations, we further sequenced 139 known deafness-related genes (Additional file 1: Table S2) by targeted NGS in 44 such probands with good quality and quantity of DNA samples.

Despite that genomic deletions containing *GJB6* and upstream regions of *GJB2* was frequently detected in several ethnic groups [14, 15], such genomic deletion was not detected in our CNV analysis based on read depth of the NGS. Instead, in three probands (D592, C290 and D1028) with mono-allelic c.235delC mutation in *GJB2*, we identified an additional heterozygous mutation c.538C > T (p. R180*) in *GJB3*, c.547G > A (p.E183K) in *GJB3* and c.228delG (p. L79Cfs*3) in *GJB6*, respectively (Table 3). These *GJB2*/*GJB3* and *GJB2*/*GJB6* mutations may combine to cause hearing loss in a digenic inheritance pattern as previously reported [15, 16].

**Table 3:** Pathogenic mutations identified by targeted NGS in probands with *GJB2* mono-allelic mutations

Patient ID	Gene ^a^	Type of variation	Nucleotide change	Amino acid change	Zygosity	Segregating with HL^b^	The *GJB2* mutant allele
D592	*GJB2*	Frameshift indel	c.235delC	p. L79Cfs*3	het	ND	c.235delC
	*GJB3*	Stop_gained	c.538C > T	p. R180*	het		
C290	*GJB2*	Frameshift indel	c.235delC	p. L79Cfs*3	het	Yes	c.235delC
	*GJB3*	Missense	c.547G > A	p. E183K	het		
D1028	*GJB2*	Frameshift indel	c.235delC	p. L79Cfs*3	het	Yes	c.235delC
	*GJB6*	Frameshift indel	c.228delG	p. W77Gfs*5	het		
D908	*STRC*	Whole gene deletion	–	–	hom	Yes	c.235delC
	*STRC*	Whole gene deletion	–	–	hom		
D2002	*STRC*	Whole gene deletion	–	–	hom	Yes	c.235delC
	*STRC*	Whole gene deletion	–	–	hom		
D1857	*STRC*	Partial gene deletion	–	–	het	Yes	c.235delC
	*STRC*	Stop_gained	c.3696G > A	p. W1232*	het		
D1807	*EYA1*	Missense	c.1276G > A	p. G426S	het	De novo	c.235delC
D281	*MITF*	Stop_gained	c.877C > T	p. R293*	het	De novo	c.299_300delAT
D349	*PCDH15*	Missense	c.4133C > T	p. T1378I	compound het	Yes	c.235delC
	*PCDH15*	Frameshift indel	c.1453delT	p. S485Rfs*2	compound het		
D554	*USH2A*	Missense	c.10904C > A	p. T3635 N	compound het	Yes	c.235delC
	*USH2A*	Missense	c.392A > G	p. N131S	compound het		
D822	*MYO15A*	Missense	c.8158G > A	p. D2720N	compound het	Yes	c.235delC
	*MYO15A*	In-frame del	c.10258_10260delTTC	p. F3420-	compound het		
C649	*CDH23*	Missense	c.7630 T > G	p. L2544 V	compound het	Yes	c.235delC
	*CDH23*	Missense	c.8257G > A	p. A2753T	compound het		
D463	*OTOF*	Stop_gained	c.2122C > T	p. R708*	compound het	Yes	c.235delC
	*OTOF*	Missense	c.1194 T > A	p. D398E	compound het		
D289	*SLC26A4*	Missense	c.1174A > T	p. N392Y	compound het	Yes	c.235delC
		Missense	c.1975G > C	p. V659 L	compound het		
D237	*SMPX*	Missense	c.55A > G	p. N19D	hemi	ND	c.235delC
D211	*TIMM8A*	Frameshift indel	c.201delT	p. E68Sfs*11	hemi	ND	c.235delC

*Abbreviations: HL* hearing loss, *het* heterozygous, *hom* homozygous, *hemi* hemizygous, *ND* not determined or not conclusive

^a^The reference sequence transcript IDs for each gene were *GJB2* NM_004004.5, *GJB3* NM_024009.2, *GJB6* NM_006783.4, *STRC* NM_153700.2, *EYA1* NM_000503.5, *MITF* NM_000248.3, *PCDH15* NM_001142771.1, *USH2A* NM_206933.2, *MYO15A* NM_016239.3, *CDH23* NM_022124.5, *OTOF* NM_194248.2, *SLC26A4* NM_000441.1, *SMPX* NM_014332.2, *TIMM8A* NM_004085.3

^b^Segregating with HL, determined on basis of segregation analysis in affected and/or unaffected family members

Our targeted NGS also identified a variety of independent pathogenic mutations in 13 (29.55%) probands (Table 3, Additional file 1: Table S3), indicating that they were simply coincidental carriers of the *GJB2* mutations. Among them, probands D908 and D2002 were found to carry homozygous deletions of the entire *STRC* gene (Additional file 1: Figure S1) and proband D1857 has a heterozygous deletion and a nonsense mutation c.3696G > A (p. W1232*) in *STRC*. Consistent with previous studies [17], all three probands with *STRC* homozygous or compound heterozygous deletions have moderate hearing loss (PTAs of 40-50 dB HL). Fourteen of the sixteen other independent mutations identified in this study have been reported to be associated with hearing loss in previous studies, including dominant mutations *EYA1* c.1276G > A (p. G426S) [18] and *MITF* c.877C > T (p. R293*) [19], recessive mutations *PCDH15* c.4133C > T (p. T1378I) and c.1453delT (p. S485Rfs*2) [20], *USH2A* c.10904C > A (p.T3635 N) [21], *MYO15A* c.8158G > A (p. D2720N) and c.10258_10260delTTC (p.F3420-) [22], *CDH23* c.7630 T > G (p. L2544 V) and c.8257G > A (p.A2753T) [20], *OTOF* c.2122C > T (p.R708*) and c.1194 T > A (p.D398E) [23, 24], *SLC26A4* c.1174A > T (p. N392Y) and c.1975G > C (p.V659 L) [25], and *SMPX* c.55A > G (p. N19D) [26]. One novel hemizygous mutation, c.201delT (p.E68Sfs*11) in *TIMM8A*, was identified in a male proband D211 as a likely pathogenic mutation since similar truncating mutations p.E24* and p.R80* in *TIMM8A* have been reported to cause hearing loss associated with Mohr-Tranebjaerg syndrome [27]. The novel c.392A > G (p.N131S) in *USH2A* identified in compound heterozygosity with the known c.10904C > A (p.T3635 N) mutation is a variant of uncertain significance (VUS).

Based on the new genetic diagnosis, we revisited the clinical aspects of proband D289 and D554. Proband D289 with *SLC26A4* c.1174A > T (p. N392Y) and c.1975G > C (p.V659 L) mutations had profound hearing loss and bilateral enlarged vestibular aqueduct, which is characteristic of biallelic *SLC26A4* mutations. Proband D554 with *USH2A* c.10904C > A (p.T3635 N) and c.392A > G (p.N131S) mutations was two years old and had no signs of retinitis pigmentosa so far. As retinitis pigmentosa may develop after puberty for patients with *USH2A* mutations, we recommended that visual acuity and visual fields of the patient be monitored by an ophthalmologist at older age.

## Discussion

In this study, we presented an overview on the mutation spectrum of *GJB2* in a large cohort (*n* = 1852) of patients with hearing loss in Chinese Hans. Bi-allelic mutations in *GJB2* are responsible for up to 25.65% of patients, representing the most frequent cause for genetic hearing loss in our cohort. The most prevalent *GJB2* mutations identified in this study were c.235delC and c.109G > A, accounting for 65.16 and 11.79% of the mutant alleles. Most (92.21%) of the patients with bi-allelic *GJB2* mutations had severe-to-profound hearing loss, in which the c.235delC is the predominant causes (84.93%). Interestingly, our results showed that c.235delC also contributes to mild-to-moderate hearing loss in a significant percentage (54.05%) of such patients, with c.109G > A being another major contributor (40.54%, Table 2). In comparison with previous studies of other Chinese ethnicities such as the Uyghur population [28], the mutation spectrum of *GJB2* is considerably different in Chinese Hans, as c.35delG, a common *GJB2* mutation in both Uyghurs and Caucasians, was detected in only one proband in our cohort.

It has long been puzzling that mutation screening of *GJB2* in a large proportion (6–15%) of patients with autosomal recessive hearing loss would identify only one pathogenic mutant allele [9, 29, 30]. In our cohort, we also identified 72 (3.89%) subjects carrying only a single recessive pathogenic mutation in *GJB2*, and that excludes those carrying the incompletely penetrant c.109G > A variant, which has a carrier frequency of 12.2% in Chinese Han normal hearing controls [31]. In our cohort, the carrier rate of mono-allelic mutations in *GJB2* (3.89% overall, 2.97% for c.235delC) is higher than that previously reported in the Chinese Han general population (2.45% overall, 1.78% for c.235delC) [32], suggesting that at least in some cases a second unidentified pathogenic mutation may act either *in cis* or *in trans* to the *GJB2* mutation to lead to the hearing loss. This hypothesis has subsequently been proved by our targeted NGS in 44 probands with mono-allelic *GJB2* mutations. In three probands, digenic inheritance of *GJB2*/*GJB3* and *GJB2*/*GJB6* mutations was identified as the likely pathogenic cause for their hearing loss (Table 3). On the other hand, two dominant and a series of recessive mutations in 11 deafness-associated genes were also identified as the independent pathogenic causes in 13 additional probands, suggesting that those probands are co-incidental carriers of the *GJB2* mutations.

Overall our targeted NGS resolved the pathogenic cause in 16 (36.36%) probands with mono-allelic *GJB2* mutations, validating the importance of high-throughput sequencing in such patients. For the remaining unresolved cases, possible pathogenic causes may include: 1) a second mutant allele in *GJB2* may exist deeply in the introns or the non-coding regulatory regions uncovered by the targeted NGS; 2) mutation in a yet unknown deafness-associated gene may lead to the hearing loss in coordination with or independent to the *GJB2* mutation; and 3) in some sporadic cases environmental factors may contribute to the hearing loss.

## Conclusions

Our results showed that mutations in *GJB2* account for over 25% of pathogenic causes in Chinese Han deaf patients, with expanded screening in other deafness-associated genes may help to further resolve cases with mono-allelic *GJB2* mutations. The sequential Sanger sequencing and targeted next generation sequencing may be an efficient approach for genetic diagnosis of deafness in Chinese Hans.

## Supplementary information

**Additional file 1: Table S1.** Phenotype and *GJB2*-associated genotypes of 1852 Chinese Han deaf patients. **Table S2.** 139 deafness-related genes sequenced in the targeted panel. **Table S3.** Function prediction of missense mutations using multiple computational tools. **Table S4.** Primers used in this study. **Figure S1.** Ratio plots showing homozygous deletion of *STRC* in patient D908, D2002 and heterozygous deletion of *STRC* in patient D1857.

## Data Availability

The data that support the findings of this study are openly available in SRA database at https://www.ncbi.nlm.nih.gov/sra, reference number PRJNA560673.
