# Supplementary material for: Molecular epidemiology of Chinese Han deaf patients with bi-allelic and mono-allelic GJB2 mutations
Source: Orphanet J Rare Dis. 2020 Jan 28;15:29. doi: 10.1186/s13023-020-1311-2 (PMC6986010; doi:10.1186/s13023-020-1311-2)
Supplement: Supplementary file 1 — Additional file 1: Table S1. Phenotype and GJB2-associated genotypes of 1852 Chinese Han deaf patients. Table S2. 139 deafness-related genes sequenced in the targeted panel. Table S3. Function prediction of missense mutations using multiple computational tools. Table S4. Primers used in this study. Figure S1. Ratio plots showing homozygous deletion of STRC in patient D908, D2002 and heterozygous deletion of STRC in patient D1857. [file 13023_2020_1311_MOESM1_ESM.docx]

**Table S1.** Phenotype and *GJB2*-associated genotypes of 1852 Chinese Han deaf patients

| Allele1 |  | Allele2 | | | Number of patients | | | | |
| --- | --- | --- | --- | --- | --- | --- | --- | --- | --- |
| Mutation | Category | Mutation | Category | | Total | Severity of hearing loss | | | |
|  |  |  |  |  |  | Mild | Moderate | Severe | Profound |
| Recessive mutations | |  |  | |  |  |  |  |  |
| Homozygous |  |  |  | |  |  |  |  |  |
| c.235delC | pathogenic | c.235delC | pathogenic | | 227 | 0 | 11 | 77 | 139 |
| p. V37I | pathogenic^*^ | p. V37I | pathogenic | | 53 | 7 | 8 | 20 | 18 |
| c.299_300delAT | pathogenic | c.299_300delAT | pathogenic | | 3 | 0 | 0 | 3 | 0 |
| c.507insAACG | pathogenic | c.507insAACG | pathogenic | | 1 | 0 | 0 | 1 | 0 |
| Compound heterozygous | |  |  | |  |  |  |  |  |
| c.235delC | pathogenic | c.299_300delAT | pathogenic | | 86 | 0 | 2 | 34 | 50 |
| c.235delC | pathogenic | p. V37I | pathogenic | | 20 | 0 | 5 | 6 | 9 |
| c.235delC | pathogenic | c.507insAACG | pathogenic | | 18 | 0 | 1 | 7 | 10 |
| c.235delC | pathogenic | p. T86R | pathogenic | | 11 | 0 | 1 | 2 | 8 |
| c.235delC | pathogenic | c.605ins46 | pathogenic | | 5 | 0 | 0 | 1 | 4 |
| c.235delC | pathogenic | p. E47* | pathogenic | | 5 | 0 | 0 | 2 | 3 |
| c.235delC | pathogenic | p. W3* | pathogenic | | 3 | 0 | 0 | 1 | 2 |
| c.235delC | pathogenic | p. R143W | pathogenic | | 3 | 0 | 0 | 1 | 2 |
| c.235delC | pathogenic | c.36insG | pathogenic | | 3 | 0 | 0 | 0 | 3 |
| c.235delC | pathogenic | p. G200R | pathogenic | | 2 | 0 | 0 | 0 | 2 |
| c.299_300delAT | pathogenic | c.176del16bp | pathogenic | | 2 | 0 | 1 | 0 | 1 |
| p. T86R | pathogenic | c.507insAACG | pathogenic | | 2 | 0 | 0 | 0 | 2 |
| c.36insG | pathogenic | c.176del16bp | pathogenic | | 2 | 0 | 0 | 0 | 2 |
| c.235delC | pathogenic | p. R75W | pathogenic | | 1 | 0 | 0 | 1 | 0 |
| c.235delC | pathogenic | p. W77* | pathogenic | | 1 | 0 | 0 | 0 | 1 |
| c.235delC | pathogenic | p. L81R | pathogenic | | 1 | 0 | 0 | 1 | 0 |
| c.235delC | pathogenic | p. T86M | pathogenic | | 1 | 0 | 0 | 0 | 1 |
| c.235delC | pathogenic | p. G130A | pathogenic | | 1 | 0 | 0 | 1 | 0 |
| c.235delC | pathogenic | c.35delG | pathogenic | | 1 | 0 | 0 | 0 | 1 |
| c.235delC | pathogenic | c.176del16bp | pathogenic | | 1 | 0 | 0 | 0 | 1 |
| c.235delC | pathogenic | c.312del14 | pathogenic | | 1 | 0 | 0 | 1 | 0 |
| c.235delC | pathogenic | p. G4D | pathogenic | | 1 | 0 | 0 | 1 | 0 |
| c.299_300delAT | pathogenic | c.-3170 G>A | pathogenic | | 1 | 0 | 0 | 1 | 0 |
| c.299_300delAT | pathogenic | p. T86R | pathogenic | | 1 | 0 | 0 | 0 | 1 |
| c.299_300delAT | pathogenic | p. R143W | pathogenic | | 1 | 0 | 0 | 0 | 1 |
| c.299_300delAT | pathogenic | c.443delC | pathogenic | | 1 | 0 | 1 | 0 | 0 |
| c.299_300delAT | pathogenic | c.605ins46 | pathogenic | | 1 | 0 | 0 | 1 | 0 |
| c.36insG | pathogenic | p. V37I | pathogenic | | 1 | 0 | 0 | 1 | 0 |
| c.36insG | pathogenic | p. R143W | pathogenic | | 1 | 0 | 0 | 1 | 0 |
| c.36insG | pathogenic | c.507insAACG | pathogenic | | 1 | 0 | 0 | 0 | 1 |
| p. W3* | pathogenic | p. W77* | pathogenic | | 1 | 0 | 0 | 1 | 0 |
| p. T86R | pathogenic | c.493insG | pathogenic | | 1 | 0 | 0 | 0 | 1 |
| p. T86R | pathogenic | c.299_300delAT | pathogenic | | 1 | 0 | 0 | 0 | 1 |
| p. R127C | pathogenic | p. G160S | pathogenic | | 1 | 0 | 0 | 0 | 1 |
| p. V37I | pathogenic | c.605ins46 | pathogenic | | 1 | 0 | 0 | 1 | 0 |
| p. V37I | pathogenic | c.299_300delAT | pathogenic | | 1 | 0 | 0 | 0 | 1 |
| p. V37I | pathogenic | p. K61Q | pathogenic | | 1 | 0 | 0 | 1 | 0 |
| p. V37I | pathogenic | p. V84M | pathogenic | | 1 | 0 | 0 | 1 | 0 |
| p. V37I | pathogenic | p. T86R | pathogenic | | 1 | 0 | 0 | 1 | 0 |
| p. V37I | pathogenic | p. R143W | pathogenic | | 1 | 0 | 0 | 1 | 0 |
| c.176del16bp | pathogenic | p. G130V | pathogenic | | 1 | 0 | 0 | 0 | 1 |
| c.176del16bp | pathogenic | p. R32H | pathogenic | | 1 | 0 | 0 | 0 | 1 |
| One recessive mutant allele only | |  |  | |  |  |  |  |  |
| p. V37I | See note | - | - | | 151 | 1 | 10 | 72 | 68 |
| c.235delC | pathogenic | - | - | | 55 | 0 | 9 | 18 | 28 |
| c.299_300delAT | pathogenic | - | - | | 8 | 0 | 1 | 2 | 5 |
| p. V63L | pathogenic | - | - | | 2 | 0 | 0 | 2 | 0 |
| p. G4D | pathogenic | - | - | | 2 | 0 | 0 | 2 | 0 |
| c.36insG | pathogenic | - | - | | 1 | 0 | 0 | 1 | 0 |
| c.507insAACG | pathogenic | - | - | | 1 | 0 | 0 | 1 | 0 |
| p. S85P | pathogenic | - | - | | 1 | 0 | 0 | 0 | 1 |
| p. T86R | pathogenic | - | - | | 1 | 0 | 0 | 1 | 0 |
| p. G130V | pathogenic | - | - | | 1 | 0 | 0 | 1 | 0 |
| Unclassified variants | |  |  | |  |  |  |  |  |
| p. T123N | unclassified | - | - | | 27 | 0 | 4 | 8 | 15 |
| p. T123N | unclassified | p. V37I | pathogenic | | 2 | 0 | 0 | 2 | 0 |
| p. T123N | unclassified | p. F191L | unclassified | | 1 | 0 | 1 | 0 | 0 |
| p. F191L | unclassified | - | - | | 1 | 0 | 0 | 0 | 1 |
| p. F191L | unclassified | p. V37I | pathogenic | | 3 | 1 | 0 | 0 | 2 |
| p. F115C | unclassified | - | - | | 3 | 0 | 0 | 1 | 2 |
| p. F115C | unclassified | p. T123N | unclassified | | 1 | 0 | 0 | 1 | 0 |
| p. T26I | unclassified | p. V37I | pathogenic | | 1 | 0 | 0 | 1 | 0 |
| p. L36P | unclassified | c.235delC | pathogenic | | 1 | 0 | 0 | 1 | 0 |
| p. A78T | unclassified | p. T86R | pathogenic | | 1 | 0 | 0 | 1 | 0 |
| p. W133S | unclassified | p. V37I | pathogenic | | 1 | 0 | 0 | 0 | 1 |
| p. V153I | unclassified | - | - | | 1 | 0 | 0 | 0 | 1 |
| p. G160S | unclassified | - | - | | 2 | 0 | 0 | 1 | 1 |
| p. R165W | unclassified | - | - | | 1 | 0 | 0 | 1 | 0 |
| p. R184P | unclassified | c.235delC | pathogenic | | 1 | 0 | 0 | 0 | 1 |
| p. M195V | unclassified | - | - | | 1 | 0 | 0 | 0 | 1 |
| p. M195V | unclassified | p. R143W | pathogenic | | 1 | 0 | 0 | 1 | 0 |
| p. I196V | unclassified | - | - | | 1 | 0 | 0 | 1 | 0 |
| p. I196T | unclassified | - | - | | 1 | 0 | 0 | 1 | 0 |
| Dominant mutations | |  |  | |  |  |  |  |  |
| p. T55N | pathogenic | - | - | | 1 | 0 | 0 | 0 | 1 |
| p. R75Q | pathogenic | - | - | | 2 | 0 | 0 | 1 | 1 |
| p. R75Q | pathogenic | p. V37I | pathogenic | | 1 | 0 | 0 | 1 | 0 |
| p. R75W | pathogenic | - | - | | 1 | 0 | 0 | 0 | 1 |
| p. R184Q | pathogenic | - | - | | 2 | 0 | 0 | 0 | 2 |
| Polymorphisms | |  |  | |  |  |  |  |  |
| p. E114G | polymorphism | - | - | | 7 | 0 | 0 | 3 | 4 |
| p. I203T | polymorphism | - | - | | 64 | 1 | 3 | 23 | 37 |
| p. V27I | polymorphism | p. V27I | polymorphism | | 16 | 0 | 1 | 4 | 11 |
| p. V27I | polymorphism | - | - | | 91 | 1 | 1 | 38 | 51 |
| p. V27I, p. E114G | polymorphism | p. E114G | polymorphism | | 78 | 0 | 5 | 30 | 43 |
| p. V27I, p. E114G | polymorphism | p. V27I | polymorphism | | 44 | 0 | 0 | 19 | 25 |
| p. V27I, p. E114G | polymorphism | p. E114G | polymorphism | | 5 | 0 | 1 | 2 | 2 |
| p. V27I, p. E114G | polymorphism | - | - | | 380 | 0 | 19 | 194 | 167 |
| p. V27I, p. E114G, p. I203T | polymorphism | - | - | | 26 | 0 | 2 | 13 | 11 |
| p. V27I | polymorphism | p. I203T | polymorphism | | 9 | 0 | 1 | 2 | 6 |
| p. E114G | polymorphism | p. I203T | polymorphism | | 1 | 0 | 0 | 1 | 0 |
| p. V207L | polymorphism | - | - | | 1 | 0 | 0 | 1 | 0 |
| No variant detected | |  |  | |  |  |  |  |  |
| - | - | - | - | | 374 | 0 | 15 | 137 | 222 |
| Total |  |  |  | 1852 | | 11 | 103 | 759 | 979 |

^*^ The p.V37I variant is a pathogenic variant with variable expressivity and incomplete penetrance

**Table S2.** 139 deafness-related genes sequenced in the targeted panel.

| Hearing loss related genes | | | | | | |
| --- | --- | --- | --- | --- | --- | --- |
| *ABHD12* | *ACTG1* | *ADGRV1* | *AIFM1* | *ALMS1* | *ANKH* | *ATP6V1B1* |
| *BCS1L* | *BSND* | *BTD* | *CABP2* | *CACNA1D* | *CCDC50* | *CDH23* |
| *CEACAM16* | *CHD7* | *CHSY1* | *CIB2* | *CISD2* | *CLDN14* | *CLRN1* |
| *COCH* | *COL11A1* | *COL2A1* | *COL4A3* | *COL4A4* | *COL4A5* | *COL4A6* |
| *COL9A1* | *COL9A2* | *COL9A3* | *DFNA5* | *DFNB59* | *DIABLO* | *DIAPH1* |
| *DSPP* | *EDN3* | *EDNRB* | *ESPN* | *ESRRB* | *EYA1* | *EYA4* |
| *FGF3* | *FOXI1* | *GAA* | *GALNS* | *GATA3* | *GIPC3* | *GJB2* |
| *GJB3* | *GJB6* | *GPSM2* | *GRHL2* | *GRXCR1* | *HARS* | *HARS2* |
| *HOXB1* | *HSD17B4* | *ILDR1* | *KARS* | *KCNE1* | *KCNJ10* | *KCNQ1* |
| *KCNQ4* | *LHFPL5* | *LOXHD1* | *LRP2* | *LRTOMT* | *MANBA* | *MARVELD2* |
| *MET* | *MITF* | *MSRB3* | *MYH14* | *MYH9* | *MYO15A* | *MYO3A* |
| *MYO6* | *MYO7A* | *NDP* | *NF2* | *NLRP3* | *OPA1* | *OTOA* |
| *OTOF* | *OTOG* | *OTOGL* | *PAX3* | *PCDH15* | *PDZD7* | *PHYH* |
| *PNPT1* | *POLR1C* | *POLR1D* | *POU3F4* | *POU4F3* | *PRPS1* | *PTPRQ* |
| *RDX* | *RMND1* | *RPS6KA3* | *SALL4* | *SEMA3E* | *SERPINB6* | *SIX1* |
| *SIX5* | *SLC17A8* | *SLC19A2* | *SLC26A4* | *SLC26A5* | *SLC29A3* | *SLC33A1* |
| *SLC52A2* | *SLC52A3* | *SMAD4* | *SMPX* | *SNAI2* | *SOX10* | *STRC* |
| *SUCLA2* | *SUCLG1* | *TBC1D24* | *TCOF1* | *TECTA* | *TFAP2A* | *TIMM8A* |
| *TJP2* | *TMC1* | *TMIE* | *TMPRSS3* | *TPRN* | *TRIOBP* | *TSPEAR* |
| *TYR* | *USH1C* | *USH1G* | *USH2A* | *WFS1* | *WHRN* |  |

**Table S3.** Function prediction of missense mutations using multiple computational tools.

| Gene | Nucleotide change | Amino acid change | Category | Function prediction | | | | | | |
| --- | --- | --- | --- | --- | --- | --- | --- | --- | --- | --- |
|  |  |  |  | SIFT  (score) | PROVEAN | Polyphen2_HDIV | Polyphen2_HVAR | MutationTaster(prob) | PANTHER | |
| *GJB2* | c.109G>A | p. V37I | Pathogenic^*^ | T (0.66) | N (-0.823) | D (1.000) | D (0.996) | D (0.999) | D |  |
| *GJB2* | c.257C>G | p. T86R | pathogenic | D (0.02) | D (-4.253) | D (1.000) | D (1.000) | D (0.999) | D |  |
| *GJB2* | c.427C>T | p. R143W | pathogenic | D (0.00) | D (-6.252) | D (1.000) | D (1.000) | D (0.999) | D |  |
| *GJB2* | c.11G>A | p. G4D | pathogenic | T (0.21) | N (-2.145) | B (0.088) | B (0.072) | N (0.999) | D |  |
| *GJB2* | c.224G>A | p. R75Q | pathogenic | D (0.00) | D (-3.997) | D (1.000) | D (1.000) | D (0.999) | D |  |
| *GJB2* | c.223C>T | p. R75W | pathogenic | D (0.00) | D (-7.994) | D (1.000) | D (1.000) | D (0.999) | D |  |
| *GJB2* | c.389G>T | p. G130V | pathogenic | D (0.00) | D (-8.602) | D (1.000) | D (0.999) | D (0.999) | D |  |
| *GJB2* | c.164C>A | p. T55N | pathogenic | D (0.00) | D (-4.690) | D (1.000) | D (1.000) | D (0.999) | D |  |
| *GJB2* | c.181A>C | p. K61Q | pathogenic | T (0.50) | N (-0.722) | P (0.877) | B (0.393) | D (0.999) | D |  |
| *GJB2* | c.187G>T | p. V63L | pathogenic | D (0.01) | D (-2.981) | D (1.000) | D (0.999) | D (0.999) | D |  |
| *GJB2* | c. 95G>A | p. R32H | pathogenic | D (0.00) | D (-4.876) | D (1.000) | D (1.000) | D (0.999) | D |  |
| *GJB2* | c.242T>G | p. L81R | pathogenic | D (0.00) | D (-5.962) | D (1.000) | D (1.000) | D (0.999) | D |  |
| *GJB2* | c.250G>A | p. V84M | pathogenic | D (0.00) | D (-2.998) | D (1.000) | D (1.000) | D (0.999) | D |  |
| *GJB2* | c.253T>C | p. S85P | pathogenic | D (0.00) | D (-3.583) | D (1.000) | D (0.986) | D (0.999) | P |  |
| *GJB2* | c.257C>T | p. T86M | pathogenic | D (0.01) | D (-3.387) | D (1.000) | D (1.000) | D (0.999) | D |  |
| *GJB2* | c.379C>T | p. R127C | pathogenic | D (0.01) | D (-3.703) | D (0.994) | B (0.423) | D (0.999) | P |  |
| *GJB2* | c.389G>C | p. G130A | pathogenic | D (0.00) | D (-5.852) | D (1.000) | D (0.995) | D (0.999) | D |  |
| *GJB2* | c.493C>T | p. R165W | pathogenic | T (0.11) | D (-5.426) | P (0.923) | B (0.298) | D (0.999) | D |  |
| *GJB2* | c.551G>A | p. R184Q | pathogenic | D (0.00) | D (-3.353) | D (1.000) | D (1.000) | D (0.999) | D |  |
| *GJB2* | c.551G>C | p. R184P | pathogenic | D (0.00) | D (-6.084) | D (1.000) | D (1.000) | D (0.999) | D |  |
| *GJB2* | c.598G>A | p. G200R | pathogenic | T (0.22) | D (-2.595) | D (0.988) | P (0.868) | D (0.999) | P |  |
| *GJB2* | c.368C>A | p. T123N | unclassified | T (0.51) | N (0.793) | B (0.000) | B (0.000) | N (0.999) | B |  |
| *GJB2* | c.571T>C | p. F191L | unclassified | D (0.00) | D (-5.719) | D (1.000) | D (1.000) | D (0.999) | D |  |
| *GJB2* | c.344T>G | p. F115C | unclassified | T (0.18) | N (0.114) | B (0.205) | B (0.264) | N (0.838) | B |  |
| *GJB2* | c.583A>G | p. M195V | unclassified | D (0.01) | D (-3.489) | D (1.000) | D (0.999) | D (0.999) | D |  |
| *GJB2* | c.77C>T | p. T26I | unclassified | D (0.01) | D (-3.357) | D (0.992) | D (0.945) | D (0.999) | P |  |
| *GJB2* | c.232G>A | p. A78T | unclassified | D (0.04) | D (-3.286) | D (0.978) | P (0.852) | D (0.999) | P |  |
| *GJB2* | c.107T>C | p. L36P | unclassified | D (0.00) | D (-4.556) | D (1.000) | D (1.000) | D (0.999) | D |  |
| *GJB2* | c.398G>C | p. W133S | unclassified | D (0.01) | D (-13.169) | D (1.000) | D (0.983) | D (0.999) | D |  |
| *GJB2* | c.457G>A | p. V153I | unclassified | T (1.00) | N (0.205) | B (0.003) | B (0.007) | D (0.816) | P |  |
| *GJB2* | c.478G>A | p. G160S | unclassified | D (0.02) | D (-2.639) | D (0.969) | P (0.557) | D (0.999) | D |  |
| *GJB2* | c.586A>G | p. I196V | unclassified | T (0.46) | N (0.144) | B (0.009) | B (0.007) | D (0.999) | P |  |
| *GJB2* | c.587T>C | p. I196T | unclassified | D (0.02) | D (-3.029) | P (0.922) | P (0.626) | D (0.999) | P |  |
| *GJB3* | c.547G>A | p. E183K | pathogenic | D (0.00) | D (-3.808) | D (1.000) | D (1.000) | D (0.999) | D |  |
| *EYA1* | c.1276G>A | p. G426S | pathogenic | D (0.00) | D (-5.565) | P (0.891) | B (0.418) | D (0.999) | D |  |
| *PCDH15* | c.4133C>T | p. T1378I | pathogenic | T (0.16) | D (-3.263) | D (1.000) | D (0.999) | D (0.999) | D |  |
| *USH2A* | c.10904C>A | p. T3635N | pathogenic | T (0.15) | N (-1.558) | P (0.631) | B (0.408) | N (0.938) | B |  |
| *USH2A* | c.392A>G | p. N131S | unclassified | T (0.42) | N (-0.984) | B (0.018) | B (0.011) | N (0.999) | P |  |
| *MYO15A* | c.8158G>A | p. D2720N | pathogenic | D (0.04) | D (-4.421) | D (0.999) | P (0.763) | D (0.999) | D |  |
| *CDH23* | c.7630T>G | p. L2544V | pathogenic | T (0.39) | N (0.626) | B (0.002) | B (0.004) | D (0.999) | - |  |
| *CDH23* | c.8257G>A | p. A2753T | pathogenic | D (0.01) | N (-1.284) | B (0.400) | B (0.120) | D (0.999) | - |  |
| *OTOF* | c.1194T>A | p. D398E | pathogenic | T (0.82) | N (-1.658) | P (0.631) | B (0.408) | D (0.998) | D |  |
| *SLC26A4* | c.1174A>T | p. N392Y | pathogenic | D (0.00) | D (-7.675) | D (1.000) | D (1.000) | D (0.999) | - |  |
| *SLC26A4* | c.1975G>C | p. V659L | pathogenic | D (0.05) | N (-1.622) | B (0.345) | B (0.417) | D (0.778) | - |  |
| *SMPX* | c.55A>G | p. N19D | pathogenic | D (0.00) | D (-4.146) | D (0.996) | D (0.986) | D (0.999) | D |  |

**Table S4.** Primers used in this study.

| Primer Name | Sequence (5’–3’) |
| --- | --- |
| *GJB2*-Exon1-F | TTTCCCAGTCTCCGAGGGAAG |
| *GJB2*-Exon1-R | AGGACGTGTGTTGGTCCAGC |
| *GJB2*-Exon2-OF | TGGTGTTTGCTCAGGAAGAG |
| *GJB2*-Exon2-OR | TGTGGCATCTGGAGTTTCAC |
| *GJB2*-Exon2-IR | ATGCTTGCTTACCCAGACTC |
| *GJB2*-Exon2-IR | TTGGGAAATGCTAGCGACTG |
| *STRC*-A-F | TGTCTTCATGCCCTCATAAATGCTT |
| *STRC*-A-R | CCAGCTGGTTTACACCTTTAGTCC |
| *STRC*-C-F | TCTTGACAAAACTAAAGCCATTGAC |
| *STRC*-C-R | CAGATTATGCCCATGCTGAAGCTA |
| *STRC*-D-F | GCTCCTCTACCCATTCAACCCAT |
| *STRC*-D-R | CTTATTTCCTTAGTAAAGAGCCGTCA |
| *STRC*-Q-OF | AAAATTAGCCAGGCATAGTGAGTGG |
| *STRC*-Q-OR | GCAGTATCCCTGGCTTCTACTCA |
| *STRC*-Q-IF | CCAGCTACTCAATAGACTGAGGTGG |
| *STRC*-Q-IR | ACTCACTAGACGCCAATAGTACCTT |
| *STRC*-c.3696-OF | GTTCAGTTCTGGCCCTACAGTTGT |
| *STRC*-c.3696-OR | CCCAAGGGCTTCAGAATCTAAGACA |
| *STRC*-c.3696-IF | TCAGTTCTGGCCCTACAGTTGTC |
| *STRC*-c.3696-IR | ATGGCTCTAGTTTCAGTTTACCACA |
| *EYA1*-F | CAAGACTGCCACCTACTGATTGACA |
| *EYA1*-R | TACTGGTGGTAATCACTTGCCTC |
| *MITF*-F | GAATCAATGAACCCGCAGTGACATTT |
| *MITF*-R | GTTCTTGCTTGATGATCCGATTCACC |
| *PCDH15*-c.4133-F | AAGACTTTCAGCCGTATTATGG |
| *PCDH15*-c.4133-R | GCAATCTCAGAGTAGTTGCACCA |
| *PCDH15*-c.1453-F | CAGACTGGTATTACTCGCTACCTCA |
| *PCDH15*-c.1453-R | CTAGATGCCTATAGTAACTACCCCTT |
| *USH2A*-c.10904-F | GTTGCAGCTACTACCCAAGGAGTT |
| *USH2A*-c.10904-R | TATATTCTTGGCTATCTTAGTCCCAT |
| *USH2A*-c.392-F | TCAGTAGCTGCATCACACCAGAC |
| *USH2A*-c.392-R | GAAAATAAATGATTCCTGCATAAACACA |
| *MYO15A*-c.8158-OF | CCAGCTTACAGATGACCCCACT |
| *MYO15A*-c.8158-OR | CCCCGAGATACCAAACAAGGC |
| *MYO15A*-c.8158-IF | GCTGCAAAATGGGACCCCTC |
| *MYO15A*-c.8158-IR | CCCCGAGATACCAAACAAGGCC |
| *MYO15A*-c.10258-F | CAGCCCAGAGAAGCTATGCAGTTC |
| *MYO15A*-c.10258-R | GGTGGGGCTCAACTCTTATCCTC |
| *CDH23*-c.7630-F | ATTGTGCAACCAAGGTAACTGAGG |
| *CDH23*-c.7630-R | TGAGACATCCTTCCCACGCTTC |
| *CDH23*-c.8257-F | ATGCTTTGCTCCCTTGAGGTTACA |
| *CDH23*-c.8257-R | GCACCAGCAGACACCCATCG |
| *OTOF*-c.2122-F | AGACCTGATTCAGAACGCAAGTGAT |
| *OTOF*-c.2122-R | GCAGACCAGCTTTGTGTGTTCCA |
| *OTOF*-c.1194-F | TCATCATTGCAGAGCACCAGTTCC |
| *OTOF*-c.1194-R | TGATTCCCAGCACTTTTACCCACT |
| *SLC26A4*-c.1174-OF | TGTCGTACAAGGACCCCAAG |
| *SLC26A4*-c.1174-OR | GAAAGGCAAATTGTCCTGCTA |
| *SLC26A4*-c.1174-IF | GCAGAGTAGGCATGGGAGTT |
| *SLC26A4*-c.1174-IR | CCTTCCTCTGTTGCCATTCC |
| *SLC26A4*-c.1975-OF | TCATCTCCTTGATGTCTTGC |
| *SLC26A4*-c.1975-OR | AAGCCCATGTATTTGCCCTG |
| *SLC26A4*-c.1975-IF | CAAGGAACAGTGTGTAGGTC |
| *SLC26A4*-c.1975-IR | TGCAATACTGGACAACCCAC |
| *SMPX*-c.55-F | GATGTACTTTTCAGACGCATTTTTCTTC |
| *SMPX*-c.55-R | CCAGGTACAGTACTTAAATTTGGCT |
| *TIMM8A*-c.201-F | GATGTACTTTTCAGACGCATTTTTCTTC |
| *TIMM8A*-c.201-R | CCAGGTACAGTACTTAAATTTGGCT |


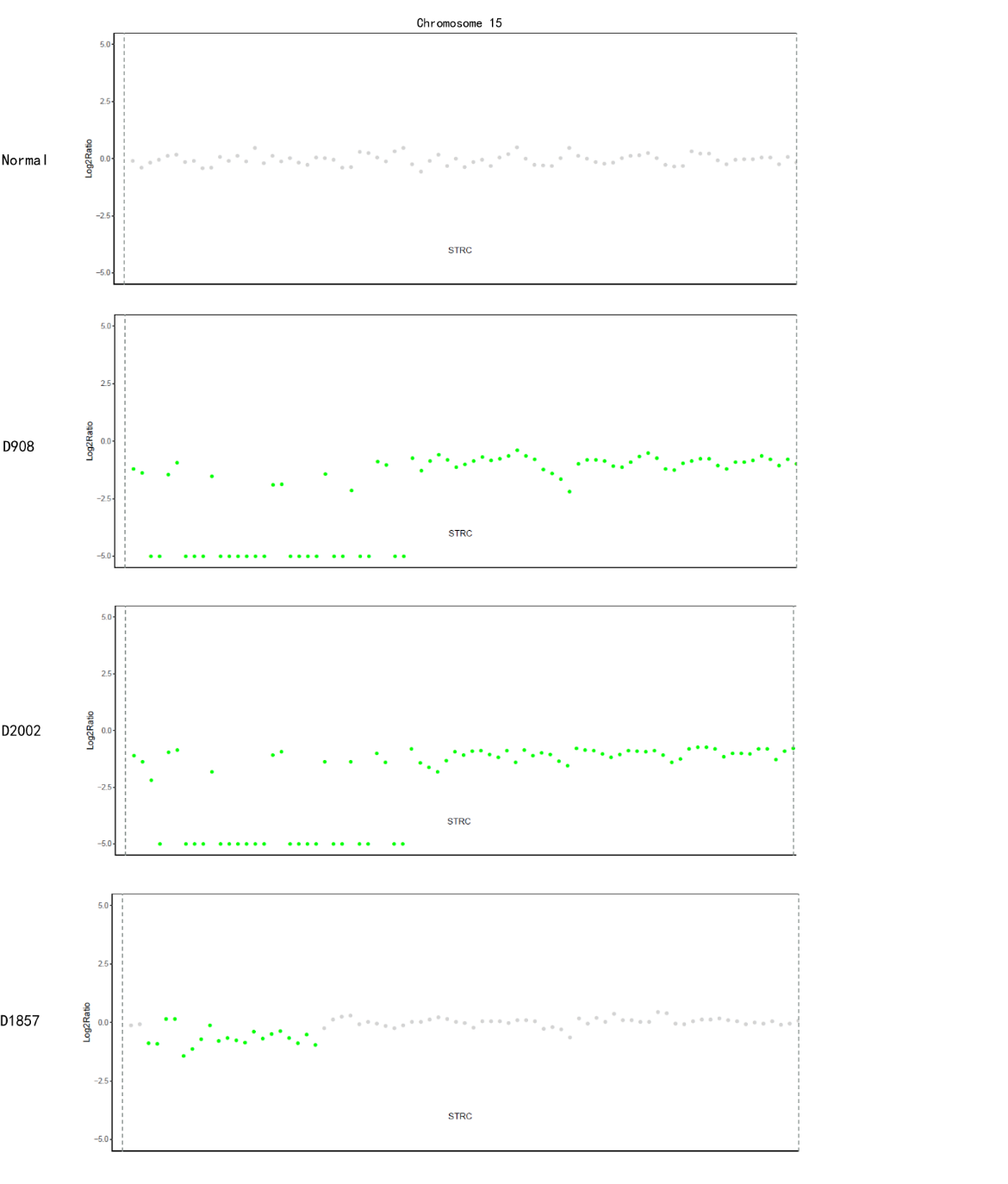


**Figure S1.** Ratio plots showing homozygous deletion of *STRC* in patient D908, D2002 and heterozygous deletion of *STRC* in patient D1857.
